# Seedling leaves allocate lower fractions of nitrogen to photosynthetic apparatus in nitrogen fixing trees than in non-nitrogen fixing trees in subtropical China

**DOI:** 10.1371/journal.pone.0208971

**Published:** 2019-03-04

**Authors:** Jingchao Tang, Baodi Sun, Ruimei Cheng, Zuomin Shi, Da Luo, Shirong Liu, Mauro Centritto

**Affiliations:** 1 Key Laboratory on Forest Ecology and Environmental Sciences of State Forestry Administration, Institute of Forest Ecology, Environment and Protection, Chinese Academy of Forestry, Beijing, China; 2 School of Environmental and Municipal Engineering, Qingdao Technological University, Qingdao, China; 3 Co-Innovation Center for Sustainable Forestry in Southern China, Nanjing Forestry University, Nanjing, China; 4 Tree and Timber Institute, National Research Council of Italy Sesto, Fiorentino, Italy; 5 Research Institute of Economic Forestry, Xinjiang Academy of Forestry Science, Urumqi, China; Tennessee State University, UNITED STATES

## Abstract

Photosynthetic-nitrogen use efficiency (PNUE) is a useful trait to characterize leaf physiology and survival strategy. PNUE can also be considered as part of ‘leaf economics spectrum’ interrelated with leaf nutrient concentrations, photosynthesis and respiration, leaf life-span and dry-mass investment. However, few studies have paid attention to PNUE of N-fixing tree seedlings in subtropical China. In this study, we investigated the differences in PNUE, leaf nitrogen (N) allocation, and mesophyll conductance (*g*_m_) in *Dalbergia odorifera* and *Erythrophleum fordii* (N-fixing trees), and *Betula alnoides* and *Castanopsis hystrix* (non-N-fixing trees). PNUE of *D*. *odorifera* and *E*. *fordii* were significantly lower than those of *B*. *alnoides* and *C*. *hystrix* mainly because of their allocation of a lower fraction of leaf N to Rubisco (*P*_R_) and bioenergetics (*P*_B_). Mesophyll conductance had a significant positive correlation with PNUE in *D*. *odorifera*, *E*. *fordii*, and *B*. *alnoides*, but the effect of *g*_m_ on PNUE was different between species. The fraction of leaf N to cell wall (*P*_CW_) had a significant negative correlation with *P*_R_ in *B*. *alnoides* and *C*. *hystrix* seedling leaves, but no correlation in *D*. *odorifera* and *E*. *fordii* seedling leaves, which may indicate that *B*. *alnoides* and *C*. *hystrix* seedling leaves did not have enough N to satisfy the demand from both the cell wall and Rubisco. Our results indicate that *B*. *alnoides* and *C*. *hystrix* may have a higher competitive ability in natural ecosystems with fertile soil, and *D*. *odorifera* and *E*. *fordii* may grow well in N-poor soil. Mixing these non-N-fixing and N-fixing trees for afforestation is useful for improving soil N utilization efficiency in the tropical forests.

## Introduction

Nitrogen (N) is very important for plants leaves, because main function of leaves- photosynthesis need a lot of N [1,2], and there was a positive correlation between photosynthetic capacity and N content in many species. However, there existed interspecific difference in the photosynthesis–N relationship [3]. Many researchers use photosynthetic-N use efficiency (PNUE, the ratio of light-saturated net CO_2_ assimilation rate (*A*_max_') to leaf N content per area (*N*_area_) [4]) to show how efficiently N resources are used during photosynthesis, and studies have been conducted on a variety of species [3,5,6]. N-fixing species could convert N from the air through legume bacteria, and always have enough N in leaves [7–9]. Studies have shown that N-fixing trees had lower *A*_max_' and higher *N*_area_, which resulted in a lower PNUE [10, 11]. These contradicting results may imply that some N-fixing species use a different strategy to utilize N compared to non-N-fixing species.

Many factors could affect PNUE, and the most important factor is leaves photosynthetic N allocation [12]. Rubisco is the most abundant enzyme in C_3_ plants [13], and it is the key factor in carbon assimilation [14]. Many researchers have found a positive correlation between leaf N fraction in Rubisco (*P*_R_) and PNUE in various plants [15–16]. Bioenergetics and the light-harvesting components could also influence PNUE in some plants [17]. Apart from photosynthetic, leaf cell walls, which could protect leave cell and influence leaf life-span also need a lot of N to synthesize [18]. Trade-offs may occur between N allocation to cell walls and Rubisco [18–20]. However, some studies have shown that these trade-offs only exist in individuals of the same species [16] or species lacking N in leaves [18, 21].

Carbon dioxide is an important raw material for photosynthesis [22], and CO_2_ partial pressure is important for Rubisco activity; this is because O_2_ is a competitive inhibitor of the C assimilatory reaction of Rubisco, promoting the Rubisco oxidation reaction [23]. A significant negative correlation between *C*_i_ (intercellular CO_2_ concentration)-*C*_c_ (CO_2_ concentration at carboxylation site) and PNUE was found in *Populus cathayana* [24]. Nitrogen is also involved in carbonic anhydrases and aquaporins [25]. These proteins play a role in mesophyll conductance (*g*_m_) by changing the nature of the diffusing molecule [26] and facilitating CO_2_ diffusion through membranes [27]. Therefore, PNUE may be influenced by *g*_m_ [25]. A significant positive correlation was found between mesophyll conductance (*g*_m_) and PNUE in six *Populus* genotypes [28].

What reason causes the low PNUE in N-fixing plants? One possible explanation is that the percentage of N in the photosynthetic apparatus is lower in the N-fixing trees [10, 11]. However, these studies neglect that *g*_m_ and the fraction of leaf N to cell wall (*P*_CW_) could also influence PNUE [19, 20, 29].We studied the factors that affect PNUE in both N-fixing and non-N-fixing large trees in a previous study and found *P*_R_ and fraction of leaf N to bioenergetics (*P*_B_) to be the main factors; the effects of *g*_m_ and *P*_CW_ were relatively small [30], but the effects in N-fixing tree seedlings remained unclear.

*Dalbergia odorifera*, *Erythrophleum fordii*, *Betula alnoides*, and *Castanopsis hystrix* are suitable for forestation in southern subtropical China and have high economic values [31–34]. *D*. *odorifera* and *E*. *fordii* are both evergreen N-fixing trees, whereas *B*. *alnoides* and *C*. *hystrix* are both non-N-fixing, and deciduous and evergreen, respectively. The objectives of our study are as follows: 1) understand how PNUE varies among *D*. *odorifera*, *E*. *fordii*, *B*. *alnoides*, and *C*. *hystrix* seedlings; 2) quantify the relationship between PNUE related to leaf N allocation and diffusional conductances to CO_2_ in seedlings.

## Materials and methods

### Study area and plant material

This study was carried out in Experimental Center of Tropical Forestry (22°7′19″–22°7′22″N, 106°44′40″–106°44′44″E) of the Chinese Academy of Forestry located in Guangxi Pingxiang, China. The location has a subtropical monsoon climate with distinct dry and wet periods where the mean annual temperature is 21°C. The mean monthly minimum and maximum temperatures are 12.1°C and 26.3°C. The mean annual precipitation is 1400 mm, and it occurs mainly from April to September. Active accumulated temperature above 10°C is 6000–7600°C. The total annual sunshine duration is 1419 hours [35,36].

Seeds of *D*. *odorifera*, *E*. *fordii*, and *C*. *hystrix* were collected from a single tree for each species, and *B*. *alnoides* seedlings were somaclone. The seeds of *D*. *odorifera*, *E*. *fordii*, and *C*. *hystrix* were germinated in a seedbed in February 2014 and *B*. *alnoides* went through budding at the same time. When the seedlings were approximately 20 cm tall, 30 similarly sized seedlings per species were individually transplanted to pots (5.4 L, filled with washed river sand) and established in an open site at the Experimental Center of Tropical Forestry in March 2014. From April to June, each pot received the same nutrient solution (0.125 g N and 0.11 g P, Hyponex M. Scott & Sons, Marysville, OH, USA) once a week, and was watered every day to keep the soil moist. Natural light (100% of light in the field) was used for illumination.

### Determination of gas exchange measurements

Gas exchange parameters were determined with a Li-6400 portable photosynthesis system (LI-COR, Lincoln, NE, USA) on sunny days from 8 am to 10 am in July and August 2014. Seven healthy and newly emerged leaves exposed to the sun in each tree species were chosen (one leaf per individual healthy tree). Photosynthetic response to photosynthetic photon flux density (PPFD) and intercellular CO_2_ concentration (*C*_i_, μmol mol^–1^) were determined for each leaf (seven repetitions in each species): Under 380 μmol mol^–1^ of leaf chamber CO_2_ concentration (the average air CO_2_ concentration in the day time), the photosynthetic rates were measured under photon flux densities of 1500, 1200, 1000, 800, 600, 400, 200, 150, 100, 80, 50, 30, 20, 10 and 0 μmol m^–2^ s^–1^ [37]. Under a saturated PPFD, the photosynthetic rates were detected using the same leaf-under leaf chamber CO_2_ concentrations of 380, 200, 150, 100, 80, 50, 380, 600, 800, 1000, 1200, 1500, 1800 and 2000 μmol mol^–1^ [28]. Relative humidity of the air in the leaf chamber was maintained at 60–70%, and leaf temperature was set at 30°C. The net photosynthetic rate (*A*_n_, μmol m^–2^ s^–1^), stomatal conductance (*g*_s_, mol CO_2_ m^–2^ s^–1^), and *C*_i_ of each sampled leaf were recorded ten times after 200 s under each PPFD and CO_2_ concentration. Then light-saturated net CO_2_ assimilation rate (*A*_max_', μmol m^–2^ s^–1^), light-saturated day respiration rate (*R*_d_, μmol m^–2^ s^–1^) and light- and CO_2_-saturated net CO_2_ assimilation rate (*A*_max_, μmol m^–2^ s^–1^) were measured or calculated. For further details see Tang *et al*. [30].

### Determination of chlorophyll fluorescence and mesophyll conductance

Fluorescence yield was measured with a Li–6400 leaf chamber fluorometer (6400–40, LI-COR, Lincoln, Nebraska, USA), using the same leaf with seven repetitions of each species. Chamber temperature was maintained at 28–32°C, and chamber air relative humidity was maintained at 60–70%. Chamber CO_2_ concentration was set to 380 μmol mol^–1^. *PPFD* was set to light saturation point. Constant values of fluorescence yield (⊿*F*/*F*_m_′) of each leaf sample were recorded 10 times after 200 s [38]. We used Loreto *et al*. [39] methods to calculate the photosynthetic electron transport rate (*J*_f_, μmol m^–2^ s^–1^):

$$J_{f}=PPFD\times \frac{\Delta F}{F_{m}\prime }\times Leafreflu\times PARDistPhotosys.$$ (1)

*Leafreflu* (leaf absorptance valued) and *PARDistPhotosys* (the fraction of quanta absorbed by photosystem II) were 0.85 [40] and 0.5 [39], respectively. We used the variable *J* method described by Harley *et al*. [41], which has been used in recent years [42–45] to calculate mesophyll conductance (*g*_m_, mol CO_2_ m^–2^ s^–1^):

$$g_{m}=\frac{A_{max}\prime }{C_{i}-\{\frac{\Gamma^{*}[J_{f}+8(A_{max}\prime +R_{d}])}{J_{f}-4(A_{max}\prime +R_{d})}\}},$$ (2)

Where *R*_d_, *C*_i_, and *A*_max_*′* were determined from gas exchange measurements. The CO_2_ photo compensation point (Γ*, μmol mol^–1^) value was 54.76 at 30°C according to Bernacchi *et al* [46].

Because the Harley method should calibrate the ETR using Chl fluorescence and gas exchange under low O_2_, we used the experience value instead (*Leafreflu* = 0.85) [30]. We also used Ethier and Livingston [47] and the exhaustive dual optimization (EDO) method [48] to calculate *g*_m_. We used software based on the Ethier and Livingston method developed by Sharkey *et al*. [49] to get *g*_m_, and uploaded our data through a website (http://www.leafweb.org) to get *g*_m_ calculated by the EDO method.

### Determination of *V*_cmax_ and *J*_max_

The mean value of *g*_m_ calculated from three methods was used to calculate CO_2_ concentration in chloroplasts (*C*_c_, μmolmol^–1^):

$$C_{C}=C_{i}-\frac{A_{max}\prime }{g_{m}}$$ (3)

Then *C*_c_ was used to fit an *A*_n_-*C*_c_ curve, followed by the maximum carboxylation rate (*V*_cmax_, μmol m^–2^ s^–1^) calculated according to Farquhar *et al*. [14], and the maximum electron transport rate (*J*_max_, μmol m^–2^ s^–1^) calculated according to Loustau *et al*. [50]. The fitting model used *in vivo* Rubisco kinetics parameters (*K*_o_, *K*_c_, and their activation energy) measured by Niinemets and Tenhunen [12].

### Analysis of quantitative limitations of photosynthetic capacity

The relative controls on photosynthetic capacity imposed by stomatal conductance (*l*_s_, %), mesophyll diffusion (*l*_m_, %), and biochemical capacity (*l*_b_, %) were calculated following the quantitative limitation analysis of Grassi and Magnani [51] as applied in Tomás *et al* [52], Peguero-Pina *et al*. [53, 54] and Nha *et al*. [55]. Different fractional limitations, *l*_s_, *l*_m_, and *l*_b_ (*l*_s_ + *l*_m_ + *l*_b_ = 1) were calculated as:

$$l_{s}=\frac{g_{tot}/g_{s}\times {\partial A}_{n}/{\partial C}_{c}}{g_{tot}+{\partial A}_{n}/{\partial C}_{c}}$$ (4)

$$l_{m}=\frac{g_{tot}/g_{m}\times {\partial A}_{n}/{\partial C}_{c}}{g_{tot}+{\partial A}_{n}/{\partial C}_{c}}$$ (5)

$$l_{b}=\frac{g_{tot}}{g_{tot}+{\partial A}_{n}/{\partial C}_{c}}$$ (6)

Where *g*_s_ and *g*_m_ were used in light-saturated and atmospheric CO_2_ concentration was 380 μmol mol^-1^, and *g*_m_ was the mean value of three methods. The *g*_tot_ is the total conductance to CO_2_ from ambient air to chloroplasts (the sum of the inverse CO_2_ serial conductances *g*_s_ and *g*_m_). The *∂A*_n_/*∂C*_c_ was calculated as the slope of *A*_n_–*C*_c_ response curves over a *C*_c_ range of 50–100 μmol mol^−1^ [53, 54].

### Determination of additional leaf traits

Leaf samples used for gas exchange measurements and leaves which size was similar to leaves used for determine photosynthesis was taken. Leaf areas were measured with a scanner (Perfection v700 Photo, Epson, Nagano-ken, Japan). Leaf dry weights were measured using an analytic balance after being oven-dried at 80°C for 48 h, then leaf mass per area (LMA, g m^-2^) was calculated.

Dried leaf samples were ground into a dry flour. Organic carbon (C) concentration was determined by the potassium dichromate-sulfuric acid oxidation method (*C*_mass_ mg g^-1^). Nitrogen concentration was determined by a VELP automatic Kjeldahl N determination apparatus (UDK-139, Milano, Italy), and leaf N per mass (*N*_mass_, mg g^-1^) and per area (*N*_area_ g m^-2^) values were calculated [30]. The PNUE (μmol mol^–1^ s^–1^) was then calculated by the formula:

$$PNUE=\frac{A_{max}\prime }{N_{area}}\times 14$$ (7)

Where 14 is the atomic mass of nitrogen.

Chlorophylls were extracted by direct immersion: 0.2 g of frozen leaves were cut into small pieces which were 5–10 mg. Leaf pieces were placed into a volumetric flask and 25 mL of 95% (v/v) alcohol was added. The flask was kept in the dark for 24 h. The absorbance of the extracts was measured at 665 nm and 649 nm with a Shimadzu visible-ultraviolet spectrophotometer (UV 2250, Fukuoka, Japan). Cell wall N content was calculated according to Onoda *et al*. [19]: 1 g of leaves were powdered in liquid N and suspended in sodium phosphate buffer (pH 7.5), the homogenate was centrifuged at 2500 g for 5 min, and the supernatant was discarded. The pellet was washed with 3% (w/v) SDS, amyloglucosidase (35 U ml^–1^, Rhizopus mold, Sigma, St Louis, MO, USA), and 0.2 M KOH, then heated and centrifuged. The pellet was then washed with distilled water and ethanol, and oven dried (75°C) for 2 days. Nitrogen in the final pellet was determined using an automatic Kjeldahl apparatus (VELP Scientifica, Usmate, Italy). The fraction of leaf N allocated to cell walls (*P*_CW_) represents the ratio of cell wall N content to the total N content.

### Calculation of N allocation in the photosynthetic apparatus

The fraction of leaf N allocated to Rubisco (*P*_R_), bioenergetics (*P*_B_), and the light-harvesting components (*P*_L_) (g g^–1^)were calculated from *V*_cmax_, *J*_max_ and chlorophyll contents using the method of Niinemets and Tenhunen [12], which has been widely used in recent years [15, 56–58]:

$$P_{R}=\frac{V_{cmax}}{6.25\times V_{cr}\times LMA\times N_{mass}}$$ (8)

$$P_{B}=\frac{J_{max}}{8.06\times J_{mc}\times LMA\times N_{mass}},$$ (9)

$$P_{L}=\frac{C_{Chl}}{C_{B}\times N_{mass}},$$ (10)

Where *C*_Chl_ is the chlorophyll concentration (mmol g^–1^), *V*_cr_ is the specific activity of Rubisco (μmol CO_2_ g^–1^ Rubisco s^–1^), *J*_mc_ is the potential rate of photosynthetic electron transport (μmol electrons μmol^–1^Cyt f s^–1^), and *C*_B_ is the ratio of leaf chlorophyll to leaf N during light-harvesting (mmol Chl (g N)^–1^). Where *V*_cr_, *J*_mc_, and *C*_B_ were calculated according to Niinemets and Tenhunen [12].The fraction of leaf N allocated to the photosynthetic apparatus (*P*_P_) was calculated as the sum of *P*_R_, *P*_B_, and *P*_L_.

### Statistical analysis

Differences between the seedling leaves were analyzed using one-way analysis of variance (ANOVA), and a post hoc test (Tukey’s test) was conducted if the differences were significant. The significance of the correlation between each pair of variables was tested with a Pearson correlation (two-tailed). All analyses were carried out using Statistical Product and Service Solutions 17.0 (SPSS17.0, Chicago, IL, USA).

## Results

### PNUE in four seedling leaves

There were significant differences in PNUE between the leaves of the four seedlings (*P <*0.001, Table 1). The PNUE in *B*. *alnoides* and *C*. *hystrix* seedling leaves were higher than those in *D*. *odorifera* and *E*. *fordii*, which was mainly attributed to their lower *N*_area_ and *N*_mass_ values. The highest PNUE in *B*. *alnoides* (120.54 μmol mol^–1^ s^–1^) was 2.6 times the lowest, found in *E*. *fordii* (45.92 μmol mol^–1^ s^–1^). However, *N*_area_ and *N*_mass_ in *B*. *alnoides* were 48.75% and 45.21% lower than in *E*. *fordii*, respectively (Table 1). There were no significant differences between *B*. *alnoides*, *C*. *hystrix*, and *D*. *odorifera* seedling leaves in *A*_max_*′* and the value in *E*. *fordii* (6.60 μmol m^–2^ s^–1^) was the smallest (Table 1). The LMA of *C*. *hystrix* (100.13 g m^-2^) was the highest (Table 1). *E*. *fordii* and *B*. *alnoides* seedling leaves had higher *C*_mass_ than *D*. *odorifera* and *C*. *hystrix*, but *C/N* was higher in *B*. *alnoides* and *C*. *hystrix* seedling leaves than *D*. *odorifera* and *E*. *fordii* (Table 1).

**Table 1:** Light-saturated photosynthesis (*A*_max_′), leaf N content per area (*N*_area_), leaf N content per mass (*N*_mass_), leaf C content per mass (*C*_mass_), C/N ratio, leaf mass per area (LMA), and photosynthetic-N use efficiency (PNUE) in seedling leaves of four species.

Leaf traits	*D*. *odorifera*	*E*. *fordii*	*B*. *alnoides*	*C*. *hystrix*	*F*
***A***_**max**_***′* (μmolm**^**–2**^**s**^**–1**^**)**	8.04±0.46^ab^	6.60±0.50^b^	8.55±1.60^a^	8.16±0.18^ab^	3.441*
***N***_**area**_ **(gm**^**-2**^**)**	2.19±0.13^a^	2.01±0.12^a^	1.03±0.25^b^	1.02±0.06^b^	36.314***
***N***_**mass**_ **(mgg**^**-1**^**)**	31.70±0.76^a^	28.09±1.49^a^	15.36±1.04^b^	10.22±0.18^c^	106.219***
***C***_**mass**_ **(mgg**^**-1**^**)**	449.50±8.86^b^	516.65±13.98^a^	493.63±5.40^a^	479.65±4.66^b^	9.713***
***C/N* (g g**^**–1**^**)**	14.24±0.48^c^	18.70±1.05^c^	32.98±2.15^b^	47.02±1.09^a^	123.492***
**LMA (gm**^**-2**^**)**	68.97±3.90^b^	71.35±0.89^b^	67.60±5.45^b^	100.13±2.60^a^	18.272***
**PNUE (μmolmol**^**–1**^**s**^**–1**^**)**	52.64±3.78^b^	45.92±2.24^b^	120.54±5.18^a^	112.01±4.62^a^	30.833***

Mean values (± SD) were shown (n = 7).

Different letters indicated significant differences between species (Tukey’s test, *P* < 0.05).

Statistically significant *F*-ratios were denoted by

*P < 0.05

**P < 0.01

***P < 0.001.

### Photosynthetic parameters in four seedling leaves

Analysis of the quantitative limitations of photosynthesis revealed that photosynthetic capacity was mainly limited by diffusional processes (*l*_s_ and *l*_m_), whereas biochemical limitations (*l*_b_) were only between 0.33% and 0.45% of the total for all studied species (Table 2).

**Table 2:** Relative stomatal (*l*_s_), mesophyll (*l*_m_) and biochemical (*l*_b_) photosynthesis limitations in four species seedling leaves.

Leaf traits	*D*. *odorifera*	*E*. *fordii*	*B*. *alnoides*	*C*. *hystrix*	*F*
***l***_**s**_ **(%)**	68.09±1.35^a^	58.23±1.93^b^	56.84±1.67^b^	56.68±2.22^b^	9.001***
***l***_**m**_ **(%)**	31.54±1.35^b^	41.39±1.95^a^	42.82±1.64^a^	42.87±2.22^a^	8.957***
***l***_**b**_ **(%)**	0.36±0.05	0.37±0.07	0.33±0.04	0.45±0.03	0.964

Mean values (± SD) were shown (n = 7).

Different letters indicated significant differences between species (Tukey’s test, *P* < 0.05).

Statistically significant *F*-ratios were denoted by

*P < 0.05

**P < 0.01

***P < 0.001.

Photosynthetic parameters were shown in Table 3 and Table 4. The *V*_cmax_ and *J*_max_ in *E*. *fordii* were higher than the other three species (Table 3) but the statistically significant values (*F*-ratios) were lower than PNUE (Table 1). Stomatal conductance (*g*_s_, 0.100 mol CO_2_ m^–2^ s^–1^) and *C*_i_ (292.88 μmol mol^–1^) in *B*. *alnoides* seedling leaves were higher than the other three species (Table 4). Moreover, *g*_m-Harley_ in *B*. *alnoides* (0.136 mol CO_2_ m^–2^ s^–1^) was higher than the other three species but *g*_m-Ethier_ (0.140 mol CO_2_ m^–2^ s^–1^) and *g*_m-Gu_ (0.160 mol CO_2_ m^–2^ s^–1^) was highest in *D*. *odorifera* (Table 4). The *C*_c_ in *B*. *alnoides* seedling leaves (all three methods) was higher than the other three species (Table 4).

**Table 3:** Maximum carboxylation rate (*V*_cmax_) and maximum electron transport rate (*J*_max_) in four species seedling leaves.

Leaf traits	*D*. *odorifera*	*E*. *fordii*	*B*. *alnoides*	*C*. *hystrix*	*F*
***V***_**cmax**_ **(μmolm**^**–2**^**s**^**–1**^**)**	78.13±4.59^b^	99.83±9.37^a^	72.98±1.51^b^	82.78±1.47^ab^	4.786**
***J***_**max**_ **(μmolm**^**–2**^**s**^**–1**^**)**	100.71±5.80^bc^	128.76±11.20^a^	98.38±5.37^bc^	109.27±4.05^ab^	3.822*

Mean values (± SD) were shown (n = 7).

Different letters indicated significant differences between species (Tukey’s test, *P* < 0.05).

Statistically significant *F*-ratios were denoted by

*P < 0.05

**P < 0.01

***P < 0.001.

**Table 4:** Stomatal conductance (*g*_s_), mesophyll conductance (*g*_m_), intercellular CO_2_ concentration (*C*_i_), and CO_2_ concentration at carboxylation site (*C*_c_) in four species seedling leaves.

Leaf traits	*D*. *odorifera*	*E*. *fordii*	*B*. *alnoides*	*C*. *hystrix*	*F*
***g***_**s**_ **(mol CO**_**2**_ **m**^**–2**^ **s**^**–1**^**)**	0.067±0.004^bc^	0.046±0.002^c^	0.100±0.013^a^	0.074±0.004^b^	11.106***
***C***_**i**_ **(μmol mol**^**–1**^**)**	251.54±6.44^b^	235.61±6.19^b^	292.88±5.94^a^	256.78±5.24^b^	7.348**
***g***_**m-Harley**_ **(mol CO**_**2**_ **m**^**–2**^ **s**^**–1**^**)**	0.114±0.013^ab^	0.068±0.007^b^	0.136±0.013^a^	0.109±0.006^ab^	15.391***
***g***_**m-Ethier**_ **(mol CO**_**2**_ **m**^**–2**^ **s**^**–1**^**)**	0.140±0.01^a^	0.066±0.01^c^	0.130±0.01^ab^	0.099±0.01^bc^	14.772***
***g***_**m-Gu**_ **(mol CO**_**2**_ **m**^**–2**^ **s**^**–1**^**)**	0.160±0.01^a^	0.063±0.01^b^	0.138±0.02^a^	0.090±0.01^b^	19.390***
***C***_**c-Harley**_ **(μmol mol**^**–1**^**)**	178.39±7.84^b^	136.80±5.18^c^	228.78±8.44^a^	172.17±6.10^b^	29.182***
***C***_**c-Ethier**_ **(μmol mol**^**–1**^**)**	192.99±7.11^b^	133.56±7.95^c^	224.91±9.13^a^	173.93±3.73^b^	27.639***
***C***_**c-Gu**_ **(μmol mol**^**–1**^**)**	200.92±6.72^a^	127.42±9.56^b^	225.58±12.27^a^	157.86±9.56^b^	20.268***

Data of CO_2_ conductance was measured in light-saturated and atmospheric CO_2_ concentration was 380 μmol mol^-1^. Mean values (± SD) were shown (n = 7).

Different letters indicated significant differences between species (Tukey’s test, *P* < 0.05).

Statistically significant *F*-ratios were denoted by

*P < 0.05

**P < 0.01

***P < 0.001.

### Leaf N allocation in four species seedling leaves

There were significant differences in leaf N allocation between the four species (*P <*0.001, Table 5). The *P*_P_ was higher than *P*_CW_ in four species seedling leaves (Table 5). The *P*_P_ was 3.9 times of the *P*_CW_ in *D*. *odorifera*, 5.4 times in *E*. *fordii*, 2.0 times in *B*. *alnoides* and 1.6 times in *C*. *hystrix*. Where *P*_R_>*P*_L_>*P*_B_ in *D*. *odorifera*, *E*. *fordii*, and *B*. *alnoides* seedling leaves, and *P*_R_>*P*_L_ = *P*_B_ in *C*. *hystrix* seedling leaves.

**Table 5:** Fraction of leaf N allocated to rubisco (*P*_R_), bioenergetics (*P*_B_), light-harvesting components (*P*_L_), photosynthetic apparatus (*P*_P_), cell wall (*P*_CW_), and other parts (1-*P*_P_-*P*_CW_, *P*_Other_) in four species seedling leaves.

Leaf traits	*D*. *odorifera*	*E*. *fordii*	*B*. *alnoides*	*C*. *hystrix*	*F*
***P***_**R**_ **(g g**^**–1**^**)**	0.13±0.01^b^	0.16±0.01^b^	0.26±0.03^a^	0.30±0.01^a^	22.130***
***P***_**B**_ **(g g**^**–1**^**)**	0.03±0.002^b^	0.04±0.003^b^	0.07±0.007^a^	0.07±0.003^a^	18.111***
***P***_**L**_ **(g g**^**–1**^**)**	0.10±0.01^ab^	0.06±0.01^c^	0.12±0.01^a^	0.07±0.01^b^	8.848***
***P***_**P**_ **(g g**^**–1**^**)**	0.27±0.02^b^	0.27±0.02^b^	0.44±0.04^a^	0.44±0.02^a^	14.796***
***P***_**CW**_ **(g g**^**–1**^**)**	0.07±0.004^c^	0.05±0.002^c^	0.22±0.010^b^	0.27±0.011^a^	182.914***
***P***_**Other**_ **(g g**^**–1**^**)**	0.66±0.02^a^	0.68±0.02^a^	0.34±0.04^b^	0.29±0.02^b^	63.830***

Mean values (± SD) were shown (n = 7).

Different letters indicated significant differences between species (Tukey’s test, *P* < 0.05).

Statistically significant *F*-ratios were denoted by

*P < 0.05

**P < 0.01

***P < 0.001.

The *P*_P_ in *B*. *alnoides* and *C*. *hystrix* seedling leaves (both were 0.44 g g^–1^) were higher than *D*. *odorifera* and *E*. *fordii* (both were 0.27 g g^–1^). The *P*_R_ and *P*_B_ in *B*. *alnoides* and *C*. *hystrix* seedling leaves were also higher than in *D*. *odorifera* and *E*. *fordii*. The *P*_L_ in *B*. *alnoides* was the highest (0.12 g g^–1^), followed by *D*. *odorifera* (0.10 g g^–1^), *C*. *hystrix* (0.07 g g^–1^), and *E*. *fordii* (0.06 g g^–1^).

### Relationship between PNUE and affecting factors

**Fig 1:**
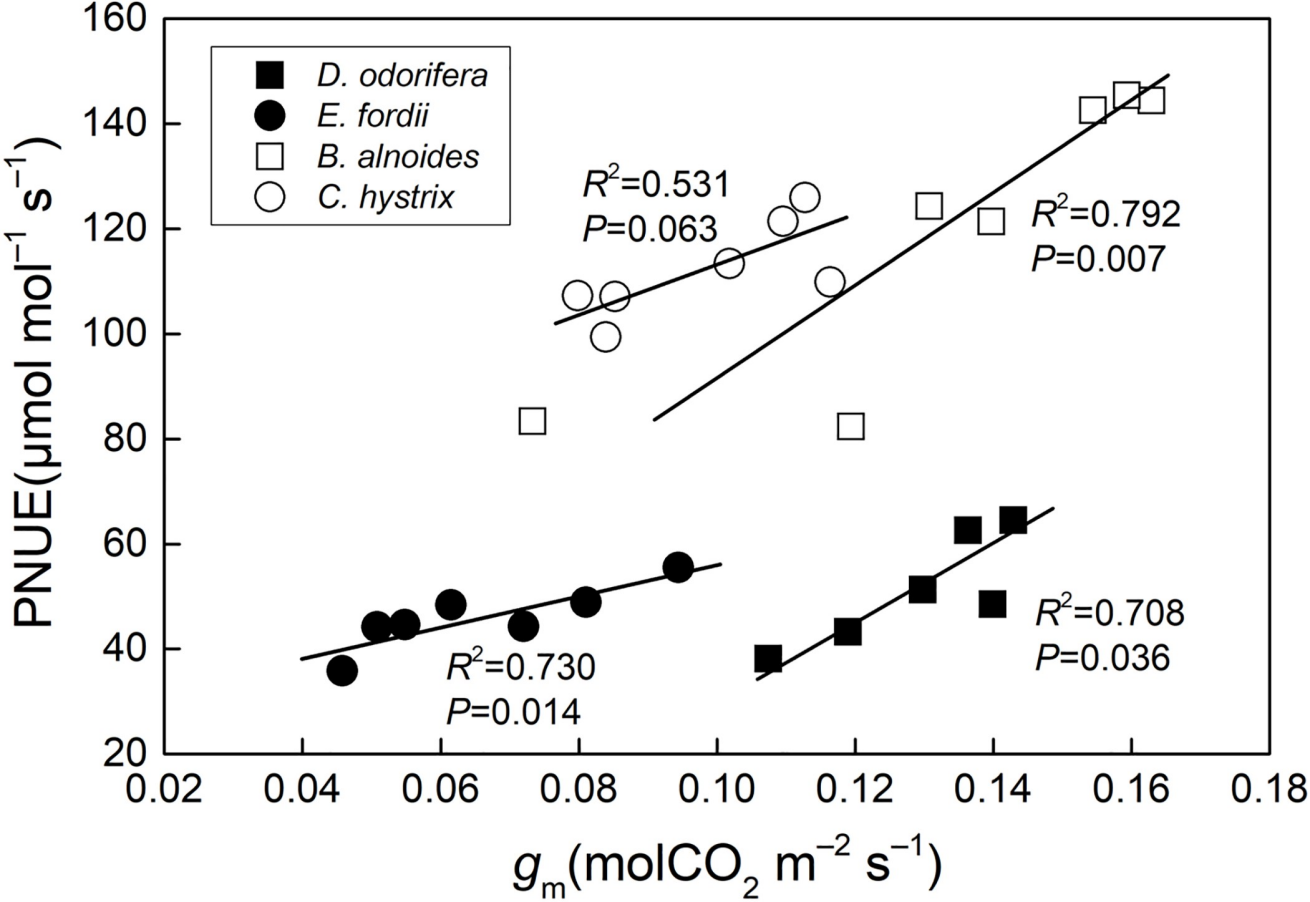
Regression analysis of mesophyll conductance (*g*_m_) with photosynthetic-N use efficiency (PNUE) in four species seedling leaves. The determination coefficient (*R*^2^) and *P*-value were also shown. The lines fitted separately for four species were significantly different (*P* < 0.05) according to the result of a one-way ANCOVA with PNUE as a dependent variable, tree species as fixed factors, and *g*_m_ as a covariate.

**Fig 2:**
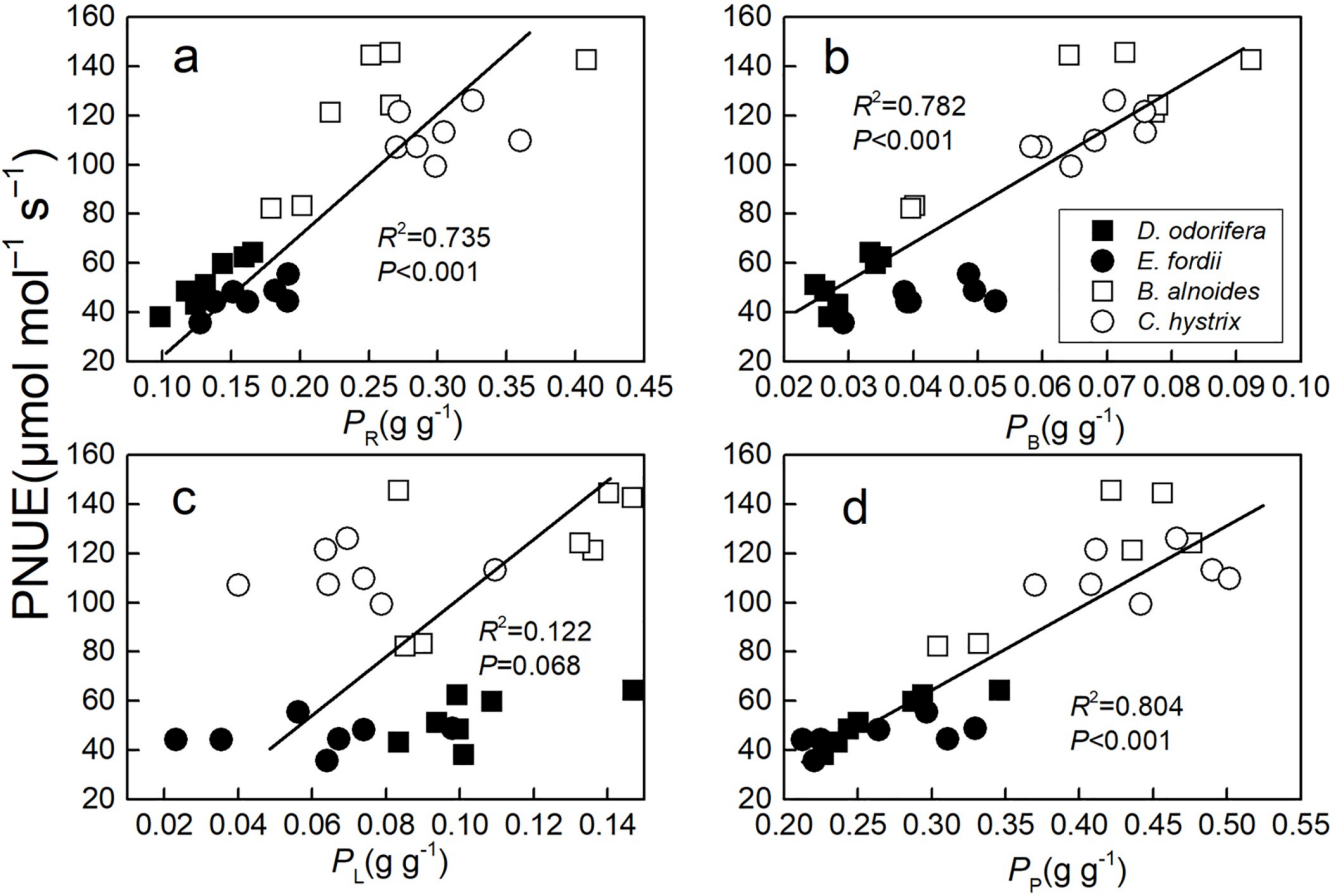
**Regression analysis of the fraction of leaf N allocated to (a) Rubisco (*P***_**R**_**), (b) bioenergetics (*P***_**B**_**), (c) light-harvesting components (*P***_**L**_**), and (d) the photosynthetic apparatus (*P***_**P**_**) with photosynthetic-N use efficiency (PNUE) in four species seedling leaves.** The determination coefficient (*R*^2^) and *P*-value were also shown. Only one line was fitted for four species, because there was no significant difference (*P* >0.05) according to the result of a one-way ANCOVA with PNUE as a dependent variable, tree species as fixed factors, and *P*_R_, *P*_B_, *P*_L,_ or *P*_P_ as a covariate.

There was a positive relationship between *g*_m_ and PNUE (*P* < 0.05), in *D*. *odorifera*, *E*. *fordii*, and *B*. *alnoides*, but not in *C*. *hystrix* (Fig 1). Both *P*_P_, *P*_R_, and *P*_B_ had a significant positive correlation with PNUE in these species (*P* < 0.001) (Fig 2).

**Fig 3:**
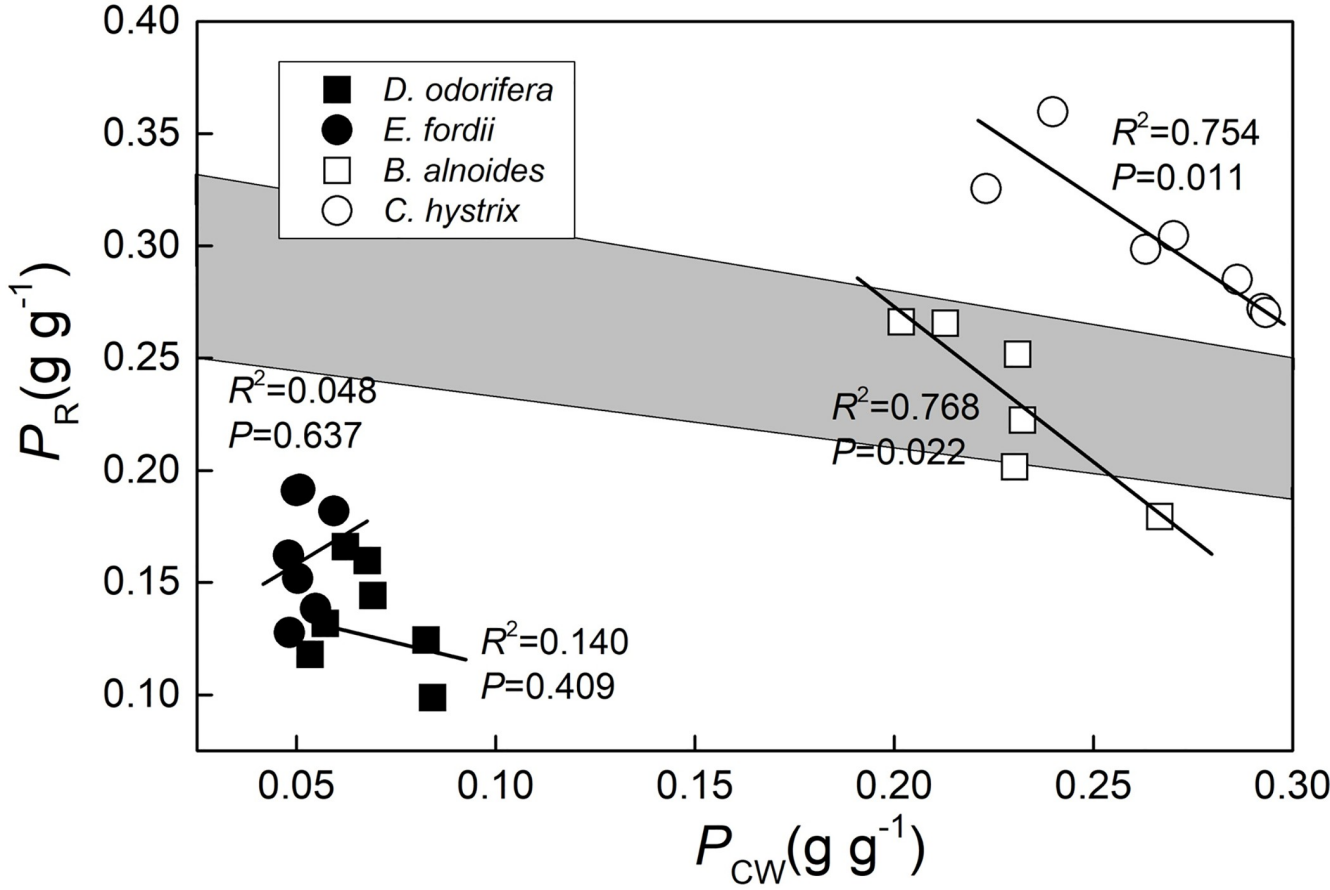
Regression analysis of the fraction of leaf N allocated to the cell wall (*P*_CW_) with leaf N allocated to Rubisco (*P*_R_) in four species seedling leaves. The determination coefficient (*R*^2^) and *P*-value were also shown. The shaded zone was drawn according to this hypothesis: when *P*_CW_ was 0.300 g g^–1^, the rest (0.700 g g^–1^) were soluble and thylakoid protein, Rubisco represented one quarter to one-third of the N in soluble and thylakoid protein, *P*_R_ valued 0.175–0.233 g g^–1^(right side of shaded zone). When *P*_CW_ was valued 0.000 g g^–1^ in limiting case (does not exist in reality), all the rest (1.000 g g^–1^) were soluble and thylakoid protein, *P*_R_ valued 0.250–0.333 g g^–1^(left side of shaded zone). For more information see Harrison *et al*. [21]. The lines fitted separately for four species were significantly different (*P* < 0.05) according to the result of a one-way ANCOVA with *P*_R_ as a dependent variable, tree species as fixed factors, and *P*_CW_ as a covariate.

The relationship between *P*_CW_ and *P*_R_ in *B*. *alnoides* (*P* = 0.022) and *C*. *hystrix* (*P* = 0.011) seedling leaves were more significant than in *D*. *odorifera* (*P* = 0.409) and *E*. *fordii* (*P* = 0.637). Regression analysis of *P*_CW_ with *P*_R_ in *B*. *alnoides* seedling leaves was within the shaded zone; *C*. *hystrix* was on the shaded zone; *D*. *odorifera* and *E*. *fordii* were under the shaded zone (Fig 3).

## Discussion

The range of PNUE in these tree seedlings was 45.92–120.54 μmol mol^–1^ s^–1^(Table 1) which was close to six *Fagus sylvatica* populations (68.74–122.22 μmol mol^–1^ s^–1^) [59] and four *Quercus* species (approximately 60–150 μmol mol^–1^ s^–1^) [20]; lower than *P*. *cathayana* (171.64–213.36 μmol mol^–1^ s^–1^) [17] and *S*. *alterniflora* (171.64–213.36 μmol mol^–1^ s^–1^) [15] under different N deposition. Wright *et al*. summed up PNUE in 710 species and the range was between 10 and 500 μmol mol^–1^ s^–1^ [60]; therefore, our results seem reasonable. Shrubs and trees usually have a low PNUE and grasses usually have high value [60]. Fast growing herbaceous species may have a PNUE higher than 200 μmol mol^–1^ s^–1^, whereas values for evergreen woody species can be lower than 50 [1]. Our values are within the medium range.

The overall result highlights a substantial difference between N-fixing and non-N-fixing tree seedling leaves in PNUE (Table 1). The variation of PNUE may be attributable to plant evolution and natural selection [61]. Low PNUE species compensate for their low productivity with a long leaf life-span [20]; stress-tolerant species [62] and late successional species [63] usually have low PNUE values. Therefore, low PNUE in *D*. *odorifera* and *E*. *fordii* may lead to high stress-tolerance traits and increase competitiveness in poor soil [64]. Higher PNUE species such as *B*. *alnoides* and *C*. *hystrix* could grow faster [20] and have a stronger competitive ability in ecosystems with fertile soil [65]. The PNUE tended to be lower for species at the ‘slow-return’ end of the leaf economics spectrum [60], and according to the ‘leaf economics spectrum’, at the slow-return end are species with long leaf life-span, expensive high-LMA leaf construction, low nutrient concentrations, and low rates of photosynthesis and respiration [4], and therefore, it can be concluded that two N-fixing species were at the ‘slow-return’ end of the leaf economics [4, 60]. Because these species live in the same area, we believe that mix these non-N-fixing and N-fixing trees for afforestation is useful for improving soil N utilization efficiency in this place.

As PNUE is the ratio of *A*_max_*′* and *N*_area_, changes of *A*_max_*′* and *N*_area_ affect PNUE. We found significant *F*-ratios in *A*_max_*′* between the four species’ seedling leaves was 3.441, lower than in *N*_area_ which was 36.314. Therefore, a change of *N*_area_ was the main reason affecting PNUE in these four species. We suspect that N-fixing species which could gain N from air by legume bacteria [7–9], may have both higher *N*_area_ and *A*_max_*′*, but our results did not support this speculation. Which reason limited *A*_max_*′* in two N-fixing species? firstly, relative stomatal (*l*_s_), and mesophyll (*l*_m_) were main reasons limited photosynthesis ability in these trees (Table 2), secondly, two N-fixing species didn’t show significant higher *g*_s_, *g*_m_, *C*_*c*_, *V*_cmax_ or *J*_max_ than non-N-fixing species (Tables 3 and 4). Therefore, we believe that a large proportion of N in the leaves of N-fixing plants did not used for photosynthesis.

N-fixing trees *D*. *odorifera* and *E*. *fordii* had significantly higher *N*_area_ than *B*. *alnoides* and *C*. *hystrix* (Table 1). Because *N*_area_ = *N*_mass_× LMA, *N*_area_ may also be affected by LMA besides N content *N*_mass_. The difference of LMA between species was far lower than the difference of *N*_mass_ (Table 1)_._ Therefore, the significantly higher *N*_mass_, caused the significantly higher *N*_area_ in *D*. *odorifera* and *E*. *fordii*. The low C/N ratio also showed high N in *D*. *odorifera* and *E*. *fordii* (Table 1). These results agreed with earlier studies [10, 11] and our study in five Fagaceae and five Leguminosae tree species [30]. However, one study reported that N-fixing trees had both higher *N*_area_ and *A*_max_*′*[66]. The relationship of *N*_area_ and *A*_max_*′*varies in different species [60], because different species have their own N allocation patterns. The N allocation in photosynthesis was more important than the total leaf N for photosynthesis [67].

Lower *P*_P_, *P*_R_, and *P*_B_ were main reasons that led to lower PNUE in N-fixing tree species (*D*. *odorifera* and *E*. *fordii*). These results agreed with previous studies [10, 11, 51] and our study on five Fagaceae tree species and five Leguminosae big tree species [30]. Rubisco catalyzes the limiting step for photosynthetic capacity [14]. A positive correlation between *A*_max_*′* and Rubisco has been frequently reported [16, 19]. An improved fraction of leaf N allocated to Rubisco could maximize the use of leaf N in photosynthesis. It should be noted that although there was a significant difference in N allocation proportion between N-fixing trees and non-N-fixing trees, there were smaller differences in N allocation quantity in Rubisco, bioenergetics, photosynthetic apparatus, cell wall, and other parts in the four species seedling leaves (mass and area, see S2 Table). The *N*_mass_ largely affected the N allocation to the photosynthetic apparatus and *P*_CW_.

The *g*_m_ could also influence the variation in PNUE through N allocation [25]. There was a significant positive relationship between *g*_m_ and PNUE in *D*. *odorifera*, *E*. *fordii*, and *B*. *alnoides*, but the effect of *g*_m_ to PNUE was not consistent between species (Fig 1). Broeckx *et al*. [28] also found this relationship in six poplar (*Populus*) genotypes, and Nha *et al*. [55] found *g*_m_ does not contribute to greater PNUE in temperate forest. We also found *g*_m_ of ten Fagaceae and Leguminosae species big trees was not significantly related to the PNUE. The effect of *g*_m_ on PNUE may also age-related.

A significant negative correlation between *P*_CW_ and *P*_R_ in *B*. *alnoides* and *C*. *hystrix* (*P* < 0.05) suggested a trade-off between N allocation to Rubisco and cell walls, whereas no trade-off was detected in *D*. *odorifera* and *E*. *fordii* (Fig 3). A similar trade-off was found in *Polygonum cuspidatum* [19], *Quercus* species [20], *Mikania micrantha* and *Chromolaena odorata* [37]; but this relationship does not exist in some other trees [16]. Some researchers believed that the main influencing factors were whether leaf N could meet the needs of both cell wall N and Rubisco N [15, 21]. We used the method described by Harrison *et al*. [21] to determine whether leaf N could meet these two needs: the regression analysis of *P*_CW_ with *P*_R_ in *B*. *alnoides* seedling leaves was within the shaded zone (the shaded zone represents the distribution area of *P*_CW_ and *P*_R_ when a trade-off exists), *C*. *hystrix* was on the shaded zone which means that *B*. *alnoides* and *C*. *hystrix* had high *P*_CW_ and *P*_R_ and therefore leaf N could not meet both needs, these two factors may affect each other. We believe the high *P*_Other_ (Table 5, possibly composed of free amino acids [68] and inorganic N (NO_3_^–^, NH_4_^+^) [69]) weakens the correlation between Rubisco and cell wall N. It must be noted that *C*. *hystrix* showed a unique relationship between *P*_CW_ and *P*_R_ (on the shaded zone), which means higher *P*_CW_ and *P*_R_ than the results of Harrison *et al*. [21]. More trees need to be studied to determine the distribution area of *P*_CW_ and *P*_R_ when a trade-off exists.

Excessive storage of N in N-fixing tree species may reduce their PNUE but may be useful for future physiological processes such as reproduction [17]. Storage of N could buffer changes in other N pools such as cell wall N [19, 20, 37] (Fig 3). Evergreen tree leaves with low PNUE have multiple roles in nutrient conservation, nutrient storage, stress tolerance, herbivore deterrence, and photosynthesis [3]. We should consider that some Rubisco can also function as N storage and may not be involve in photosynthesis [70, 71]. This type of Rubisco might lead to greater rates of photosynthesis under suboptimal conditions [3]. Therefore, Rubisco N calculated by the model of Farquhar *et al*. [14] might be N in activated Rubisco. Using chemical methods to extract and determine Rubisco N content could be useful [20, 72].

We used to do experiment with *C*. *hystrix* big trees, and its PNUE was 74.34±8.54 μmol mol^–1^ s^–1^ [30], smaller than its seedlings (Table 1). *C*. *hystrix* big tree also had higher *P*_CW_ (0.46 g g^-1^) than seedlings, but its *P*_P_ (0.26 g g^-1^), *P*_R_ (0.20 g g^-1^), *P*_B_ (0.041 g g^-1^) and *P*_L_ (0.014 g g^-1^) [30] were lower than seedlings (Table 1). Seedlings with high PNUE could grow fast, reach the canopy earlier and increased ability of competition for light [5]. Big trees with higher *P*_CW_ could better resist the environmental stress in canopy, such as typhoon, insect attack and diseases [19]. We suspect that trade-offs for N allocation to photosynthesis versus cell walls may also exist at different stages of a tree's growth, in order to meet the N demand in different growth stages.

Although both the *B*. *alnoides* and *C*. *hystrix* are non-N-fixing broadleaf plants, and have some similar functional traits, there were significant differences showed in *N*_mass_, LMA, *g*_m_, *C*_c_, and *P*_CW_ (Tables 1, 4 and 5). *B*. *alnoides* is a deciduous broad-leaved plant and *C*. *hystrix* is an evergreen broad-leaf plant. In order to contribute to a longer leaf life span, evergreen broad-leaf plants should improve leaf tolerance to environmental disturbance [73,74], reflected in higher LMA [60], and *P*_CW_ [16]. Higher defensive investment could also reduce *N*_mass_ [16]. Simultaneously, if higher LMA is a result of mesophyll cell wall thickening, it will reduce *g*_m_ and *C*_c_ [75, 76], and variations in LMA are often inversely correlated with *g*_m_ and *C*_c_ [77, 78], consistent with the results of those two species.

## Conclusions

This study indicated that PNUE was significantly lower in two N-fixing trees (*D*. *odorifera* and *E*. *fordii*) than that in two non-N-fixing trees (*B*. *alnoides* and *C*. *hystrix*). This finding was mainly attributed to lower *P*_R_ and *P*_B_. *B*. *alnoides* and *C*. *hystrix* optimized their leaf N allocation to photosynthesis. Although *g*_m_ had a significant positive correlation with PNUE in *D*. *odorifera*, *E*. *fordii*, and *B*. *alnoides*, the effect of *g*_m_ on PNUE was different between species. *P*_CW_ had a significant negative correlation with *P*_R_ in *B*. *alnoides* and *C*. *hystrix* seedling leaves, but there was no significant correlation between *P*_CW_ and *P*_R_ in *D*. *odorifera* and *E*. *fordii* seedling leaves, which may indicate that *B*. *alnoides* and *C*. *hystrix* seedling leaves did not have enough N to satisfy the demand from both the cell wall and Rubisco. *B*. *alnoides* and *C*. *hystrix* with higher PNUE may have a higher competitive ability in natural ecosystems with fertile soil. Our results indicate that mixing these non-N-fixing and N-fixing trees for afforestation is useful for improving soil N utilization efficiency in the tropical forests.

## Supporting information

S1 Table Chlorophyll contents (chlorophyll a, chlorophyll b, chlorophyll a+b and Chla/b) in four species seedling leaves. Mean values (± SD) were shown (n = 7). Different letters indicated significant differences between species (Tukey’s test, *P*<0.05). Statistically significant *F*-ratios were denoted by *P<0.05, **P<0.01, ***P<0.001.
(DOCX)

S2 Table Quantity of leaf N (per area and per mass) allocated to Rubisco (*Q*_Rarea,_ *Q*_Rmass_), bioenergetics (*Q*_Barea,_ *Q*_Bmass_), light-harvesting components (*Q*_Larea,_ *Q*_Lmass_), photosynthetic apparatus (*Q*_Parea,_ *Q*_Pmass_), cell wall (*Q*_CWarea,_ *Q*_CWmass_), and other parts (*Q*_Other-area,_ *Q*_Other-mass_) in four species seedling leaves. Mean values (± SD) were shown (n = 7). Different letters indicated significant differences between species (Tukey’s test, *P*<0.05). Statistically significant *F*-ratios were denoted by *P<0.05, **P<0.01, ***P<0.001.
(DOCX)
