# Supplementary material for: Seedling leaves allocate lower fractions of nitrogen to photosynthetic apparatus in nitrogen fixing trees than in non-nitrogen fixing trees in subtropical China
Source: PLoS One. 2019 Mar 4;14(3):e0208971. doi: 10.1371/journal.pone.0208971 (PMC6398865; doi:10.1371/journal.pone.0208971)
Supplement: S1 Table — Mean values (± SD) were shown (n = 7). Different letters indicated significant differences between species (Tukey’s test, P<0.05). Statistically significant F-ratios were denoted by *P<0.05, **P<0.01, ***P<0.001. (DOCX) [file pone.0208971.s001.docx]

**Table S1. Chlorophyll contents (chlorophyll a, chlorophyll b, chlorophyll a+b and Chla/b) in four species seedling leaves.**

| **Leaf traits** | ***D. odorifera*** | ***E. fordii*** | ***B. alnoides*** | ***C. hystrix*** | ***F*** |
| --- | --- | --- | --- | --- | --- |
| **Chl*a* (mg g^-1^)** | 1.33±0.03^a^ | 0.75±0.11^b^ | 0.93±0.01^b^ | 0.35±0.04^b^ | 45.418^***^ |
| **Chl*b* (mg g^-1^)** | 1.05±0.03^a^ | 0.56±0.09^b^ | 0.70±0.01^b^ | 0.33±0.04^c^ | 34.791^***^ |
| **Chl*a+b* (mg g^-1^)** | 2.38±0.05^a^ | 1.31±0.20^b^ | 1.63±0.03^b^ | 0.68±0.08^c^ | 40.548^***^ |
| **Chl*a/b*** | 1.27±0.05^a^ | 1.34±0.07^a^ | 1.33±0.08^a^ | 1.06±0.05^b^ | 28.531^***^ |

Mean values (± SD) were shown (n = 7). Different letters indicated significant differences between species (Tukey’s test, *P*<0.05). Statistically significant *F*-ratios were denoted by ^*^ *P*<0.05, ^**^ *P*<0.01, ^***^ *P*<0.001.
