# Supplementary material for: Seedling leaves allocate lower fractions of nitrogen to photosynthetic apparatus in nitrogen fixing trees than in non-nitrogen fixing trees in subtropical China
Source: PLoS One. 2019 Mar 4;14(3):e0208971. doi: 10.1371/journal.pone.0208971 (PMC6398865; doi:10.1371/journal.pone.0208971)
Supplement: S2 Table — Mean values (± SD) were shown (n = 7). Different letters indicated significant differences between species (Tukey’s test, P<0.05). Statistically significant F-ratios were denoted by *P<0.05, **P<0.01, ***P<0.001. (DOCX) [file pone.0208971.s002.docx]

**Table S2. Quantity of leaf N (per area and per mass) allocated to Rubisco** **(*Q*_Rarea,_ *Q*_Rmass_), bioenergetics (*Q*_Barea,_ *Q*_Bmass_), light-harvesting components (*Q*_Larea,_ *Q*_Lmass_), photosynthetic apparatus (*Q*_Parea,_ *Q*_Pmass_), cell wall (*Q*_CWarea,_ *Q*_CWmass_), and other parts (*Q*_Other-area,_ *Q*_Other-mass_) in four species seedling leaves.**

| **Leaf traits** | ***D. odorifera*** | ***E. fordii*** | ***B. alnoides*** | ***C. hystrix*** | ***F*** |
| --- | --- | --- | --- | --- | --- |
| ***Q*_Rarea_ (g m^-2^)** | 0.288±0.012^ab^ | 0.330±0.029^a^ | 0.250±0.006^b^ | 0.308±0.010^ab^ | 4.054^*^ |
| ***Q*_Rmass_ (mg g^-1^)** | 4.24±0.24^a^ | 4.61±0.39^a^ | 3.85±0.34^ab^ | 3.08±0.10^b^ | 5.119^**^ |
| ***Q*_Barea_ (g m^-2^)** | 0.064±0.003^b^ | 0.085±0.008^a^ | 0.064±0.004^b^ | 0.069±0.002^ab^ | 4.384^*^ |
| ***Q*_Bmass_ (mg g^-1^)** | 0.94±0.03^ab^ | 1.19±0.10^a^ | 0.99±0.10^ab^ | 0.69±0.04^b^ | 7.021^**^ |
| ***Q*_Larea_ (g m^-2^)** | 0.224±0.010^a^ | 0.117±0.017^b^ | 0.116±0.009^b^ | 0.073±0.008^c^ | 29.689^***^ |
| ***Q*_Lmass_ (mg g^-1^)** | 3.32±0.25^a^ | 1.64±0.24^b^ | 1.72±0.07^b^ | 0.73±0.09^c^ | 34.689^***^ |
| ***Q*_Parea_ (g m^-2^)** | 0.577±0.019^a^ | 0.532±0.042^a^ | 0.430±0.011^b^ | 0.450±0.015^b^ | 7.820^**^ |
| ***Q*_Pmass_ (mg g^-1^)** | 8.50±0.46^a^ | 7.44±0.56^ab^ | 6.57±0.45^b^ | 4.51±0.18^c^ | 15.163^***^ |
| ***Q*_CWarea_ (g m^-2^)** | 0.150±0.015^b^ | 0.103±0.005^b^ | 0.230±0.029^a^ | 0.273±0.013^a^ | 18.677^***^ |
| ***Q*_CWmass_ (mg g^-1^)** | 2.16±0.16^b^ | 1.44±0.06^c^ | 3.39±0.29^a^ | 2.73±0.11^ab^ | 22.224^***^ |
| ***Q*_Other-area_ (g m^-2^)** | 1.459±0.114^a^ | 1.372±0.099^a^ | 0.372±0.068^b^ | 0.299±0.023^b^ | 56.309^***^ |
| ***Q*_Other-mass_ (mg g^-1^)** | 21.05±0.86^a^ | 19.20±1.27^a^ | 5.42±0.88^b^ | 2.99±0.22^b^ | 108.988^***^ |

Mean values (± SD) were shown (n = 7). Different letters indicated significant differences between species (Tukey’s test, *P*<0.05). Statistically significant *F*-ratios were denoted by ^*^ *P*<0.05, ^**^ *P*<0.01, ^***^ *P*<0.001.
